# The Ethanol Extract of Avocado (*Persea americana* Mill. (Lauraceae)) Seeds Successfully Induces Implant Regression and Restores Ovarian Dynamic in a Rat Model of Endometriosis

**DOI:** 10.1155/2020/8521831

**Published:** 2020-07-25

**Authors:** Stéphane Minko Essono, Marie Alfrede Mvondo, Esther Ngadjui, François Xavier Kemka Nguimatio, Pierre Watcho

**Affiliations:** Animal Physiology and Phytopharmacology Laboratory, University of Dschang, P.O. Box 67, Dschang, Cameroon

## Abstract

Endometriosis is an estrogen-dependent disease with conventional therapies which do not have desirable effectiveness and possess many side effects. Scientific evidences suggest that medicinal plants with antioxidant, anti-inflammatory, and/or antiproliferative properties are potential alternatives for the treatment of endometriosis. The ethanol extract of *Persea americana* Mill. (Lauraceae) seeds was found exhibiting antiproliferative properties *in vitro* and *in vivo*. This study therefore is aimed at investigating the effects of such an extract on an experimental model of endometriosis. Endometriosis was induced by grafting uterine fragments onto the peritoneum of female Wistar rats. After checking the success of the transplantation surgery, animals with endometriosis were orally treated with the ethanol extract of *P. americana* seeds at the doses of 12.5, 25, and 50 mg/kg. The positive control was treated with letrozole (10 mg/kg) while the negative control received the vehicle. Treatments lasted 7 days and animals were sacrificed thereafter. Endometrial implant volume was determined. Estradiol and progesterone levels were measured in serum samples and endometriosis lesions. The oxidative status of endometriosis lesions was evaluated. Histological analysis of endometriosis lesions, uterus, and ovaries was also performed. Results showed that the ethanol extract of *P. americana* seeds decreased endometrial implant volume (*p* < 0.001) and serum levels of estradiol and progesterone (*p* < 0.01). The levels of estradiol also decreased in endometriosis lesions at doses of 12.5 and 50 mg/kg (*p* < 0.001). Both malondialdehyde and glutathione levels increased in endometriosis lesions (*p* < 0.001). The ectopic endometrium height decreased and the number of antral follicles and corpora lutea (*p* < 0.05) increased while that of luteinized unruptured follicles decreased (*p* < 0.001). In conclusion, the ethanol extract of *P. americana* seeds displayed an antiendometriosis effect suggesting that it could be a potential alternative for the treatment of endometriosis.

## 1. Introduction

Endometriosis is commonly defined as the development of endometrial tissue, consisting of both glandular epithelium and stroma outside the uterine cavity [1]. This gynecological condition affects approximately 10–14% of reproductive-aged women with symptoms like dysmenorrhea, dyspareunia, and infertility [2]. Although theories of coelomic metaplasia, altered immunity, stem cells, genetics, and retrograde menstruation have been proposed to explain the development of the disease [3], the pathogenesis of endometriosis is still unknown. In fact, the theory of retrograde menstruation proposed in 1927 by Sampson [4] appears to be the most well accepted hypothesis. According to this theory, during menses, there is a retrograde migration of blood along the uterine horn into the peritoneal cavity, where it adheres to the peritoneal surface, invades extracellular matrix, and initiates angiogenesis [5]. In addition, the proliferation process observed in endometriosis is due to high estrogen levels which constantly stimulate the growth of endometrial lesions, reduced antioxidant enzymes like glutathione (GSH), and increased resistance of endometriotic cells to progesterone stimulation [6], as progesterone was reported to convert estradiol to estrone in the normal endometrium and to therefore reduce estradiol stimulation [6]. Moreover, it has been reported that the active estrogen (estradiol (E_2_)) is a major contributor to developmental angiogenesis of endometriotic tissue [7]. Indeed, estrogen was found activating the vascular endothelial growth factor (VEGF), which stimulates angiogenesis on implanted tissue [7]. On the other hand, E_2_, estrone, and aromatase were reported to be produced by the endometriosis lesions [8]. In this regard, it has been demonstrated that the overexpression of aromatase occurs in endometriotic tissue and its activity is significantly higher than that of the normal endometrium [9]. Therefore, the strong expression of aromatase as a main enzyme involved in estrogen synthesis results in considerably higher E_2_ synthesis, which is responsible for cellularity in endometriosis lesions.

The gold standard for the diagnosis of peritoneal endometriosis is a visual inspection by laparoscopy followed by histological confirmation [10]. Treatment options for endometriosis include nonsteroidal anti-inflammatory drugs (NSAIDs), hormones (oral contraceptives (OCPs) and GnRH analogues), and surgery [1]. However, these treatments are relatively expensive and have several side effects [11]. NSAIDs, for instance, only modulate pain, as the disease continues to progress [12]. GnRH analogues may induce early menopause with its associated symptoms [13]. In addition, oral contraceptives were reported to increase the risk of breast and brain carcinoma [12]. These limitations of existing treatments led to an increase interest in researching new alternatives and less harmful treatments for endometriosis. The literature suggests that medicinal plants with antiproliferative, anti-inflammatory, and antioxidative activities are potential alternatives for the treatment of endometriosis [14–16].

*Persea americana* Mill. (Lauraceae), for instance, is a fruit tree widespread in tropical and subtropical regions. This tree is also of particular importance nutritionally through its fruits which are often used in salads with very high fat content, mainly unsaturated fatty acids. *P. americana* fruits are commonly known as “avocados.” Nowadays, a likely anticancer activity of *P. americana* (avocado) seeds, most often thrown away, has generated growing scientific interest. Indeed, the ethanol extract of *P. americana* seeds was found stimulating apoptosis of Jurkat lymphoblastic cells [17]. The cytotoxic effect of the same extract was also reported on breast cancer cell line (MCF-7) and human liver cancer cell line (HepG2) [18]. In addition, Ding et al. reported that *P. americana* extract selectively induces apoptosis of cancer cells but not normal cells [19]. Recently, a study carried out by our research team showed that the ethanol extract of *P. americana* seeds prevented tamoxifen-induced endometrial hyperplasia in female Wistar rats [20]. Although carrying all these properties, the ethanol extract of avocado seeds has not yet been investigated on endometriosis. The present study therefore aimed at evaluating the ability of an ethanol extract of avocado seeds to inhibit endometriosis implant growth in female Wistar rats. The measurement of ovarian hormones (estradiol and progesterone) and oxidative stress allowed elucidating the probable mechanism of action of this extract. The histological analysis of the ovaries allowed determining whether or not this extract restored ovarian folliculogenesis which is reported to be disrupted by endometriosis [21, 22].

## 2. Materials and Methods

### 2.1. Chemicals Substances

Estradiol valerate (Progynova^®^ 2 mg) was purchased from Delpharm (Lille, France) and letrozole (2.5 mg) from Denk Pharma (Munchen, Germany).

### 2.2. Plant Collection and Preparation of the Ethanol Extract

The same variety of avocados was collected from the same tree in Dschang (West Region, Cameroon) in June 2019. The authentication of the plant was done using leaves, fruits, and flowers of the avocado tree (*Persea americana*) in comparison with the botanical sample N°80 of Daniel Dong registered at the Cameroon National Herbarium, where a voucher specimen has been deposited under the number 18604/SFR/CAM.

Fresh avocado seeds were grated and the powder obtained was directly shade-dried. Six kilograms of the dried powder of avocado seeds were macerated in 10 L of ethanol 95% for 72 h at room temperature. The resulting solution was filtered with Whatman paper number 4 and then evaporated to dryness using a rotary evaporator at 79°C; from this process, 30.54 g of ethanol extract was obtained (extraction yield: 0.51%). This extract was kept in an airtight container at 4°C until used.

### 2.3. Justification of the Doses Used

Letrozole was administered at the dose of 10 mg/kg. This dose was chosen based on the work of Pritts et al. [23] who reported that this dose of letrozole induced ovulation and controlled ovarian hyperstimulation in women with oligoanovulation. Three doses (12.5, 25, and 50 mg/kg) of the ethanol extract of *P. americana* seeds were administered to rats. At these doses, the ethanol extract of *P. americana* seeds was found to prevent tamoxifen-induced endometrial hyperplasia in rats [20].

### 2.4. Animals

Adult female Wistar rats weighing 170–200 g were obtained from the breeding facility of the Research Unit of Animal Physiology and Phytopharmacology, University of Dschang (Cameroon). All rats had diet and water *ad libitum*. Animals handling was carried out after the approval of the research proposal by the scientific committee of the Department of Animal Biology, University of Dschang, in conformity with the European community guidelines (EEC Council Direction 2010/63/EU of 22 September 2010) [24].

### 2.5. Study Design

#### 2.5.1. Endometriosis Induction

The method described by Pereira et al. [25] was used to induce endometriosis. Briefly, thirty adult female Wistar rats were anesthetized with Diazepam (10 mg/kg: i.p.) and Ketamine (50 mg/kg: i.p.) and subjected to a 2.5 cm longitudinal suprapubic incision starting 0.5 cm above the pubis. A tunnel was made between the abdominal wall and the subcutaneous tissue, and an incision was performed on the right inguinal region. The abdominal wall was opened; the left uterus was located, ligatured, and excised. The excised uterine horn was opened longitudinally and two fragments of 1 cm each were retrieved and grafted onto the muscle in the right inguinal region using sewing thread. Finally, the abdominal wall was closed with two layers sutured using the same thread. Following induction, animals received on days 5, 9, and 13, estradiol valerate at the dose of 10 mg/kg to promote the growth of the ectopic endometrium. Sham-operated animals were subjected to the same procedure without transplantation of uterine fragments. Three weeks later, animals underwent an exploratory laparotomy to examine if peritoneal endometriosis lesions had been successfully established and to determine their volume as previously described [16, 26].

#### 2.5.2. Treatments and Sacrifice

After a recovery period of 14 days, animals were assigned to one of the following treatment groups (*n* = 5 animals per group):

- NORMAL: normal animals receiving distilled water (10 ml/kg)
- SHAM: sham-operated animals receiving distilled water (10 ml/kg)
- [ENDO + DW]: animals with endometriosis receiving distilled water (10 ml/kg)
- [ENDO + LTZ]: animals with endometriosis receiving letrozole at the dose of 10 mg/kg
- [ENDO + EE12.5]: animals with endometriosis receiving the ethanol extract of *P. americana* seeds at the dose of 12.5 mg/kg
- [ENDO + EE25]: animals with endometriosis receiving the ethanol extract of *P. americana* seeds at the dose of 25 mg/kg
- [ENDO + EE50]: animals with endometriosis receiving the ethanol extract of *P. americana* seeds at the dose of 50 mg/kg

Treatments were given orally for 7 consecutive days and animals were subjected to a 12-hour fast thereafter. This treatment period was extrapolated from the observations of Pritts et al. [27] who reported the ability of letrozole to induce ovulation following a 5-day treatment period and to control hyperstimulation in women with ovulation dysfunction. After 12 hours of fasting, animals were sacrificed under anesthesia (Diazepam/Ketamine). The abdominal cavity was opened and the volume of ectopic foci (endometrial implants) was determined again. Ectopic foci were then excised and homogenized in 0.9% saline solution (0.1 g per 1 mL). Tissue homogenates were centrifuged at 3000 rpm for 15 minutes at 5°C. The resulting supernatants were collected and stored at −20°C for biochemical analysis. Blood was also collected from each rat by catheterization of the abdominal artery and centrifuged at 3000 rpm for 15 minutes and the supernatant was kept at −20°C for biochemical analysis. The uterus and ovaries were collected, cleaned of fat tissues, weighed, and fixed in 10% formalin for histological analysis. Part of each collected endometrial implant was also fixed in 10% formalin for histological analysis.

### 2.6. Biochemical Analysis

#### 2.6.1. Oxidative Stress

Malondialdehyde (MDA) levels in endometrial implants were determined by the method of Wilbur et al. [28] which is based on the reaction with the thiobarbituric acid (TBA) at 90°C–100°C. In the TBA test, MDA reacts with the production of a pink pigment having an absorption maximum at 532 nm. The following formula was used to determine tissue levels of malondialdehyde:

(1) $$\begin{array}{c} \left[\text{MDA}\right]\;=\frac{\mathrm{\Delta DO}}{\mathrm{\varepsilon .l.m.}}, \end{array}$$

where [MDA] is concentration of malondialdehyde (nM/mg of tissue), ΔDO is absorbance of the sample absorbance of the reagent blank, *ε* is molar extinction coefficient (1.56.10^−4^ nM^−1^·cm^−1^), l is the length of path (1 cm), and *m* is the mass of tissue collected for the homogenization (mg).

Glutathione (GSH) levels in endometrial implants were determined by the method of Sehirli et al. [29]. GSH is oxidized by 5,5′-dithiobis-2-nitrobenzoic acid (DTNB). This results in the formation of a yellow compound, the 5-thio-2-nitrobenzoic acid (TNB), having an absorption maximum at 412 nm. Tissue levels of GSH were determined as follows:

(2) $$\begin{array}{c} \left[\text{GSH}\right]=\frac{\Delta \text{DO}}{\varepsilon \text{.l.m.}}, \end{array}$$

where [GSH] is concentration of GSH (nM/mg of tissue), ΔDO is absorbance of the sample absorbance of the reagent blank, *ε* is molar extinction coefficient (1.36.10^−5^ nM^−1^·cm^−1^), l is path length (1 cm), and *m* = mass of the tissue collected for homogenization (mg).

#### 2.6.2. Estradiol and Progesterone

Serum and tissue (endometrial implants) levels of estradiol and progesterone were assessed by ELISA tests using reagent kits purchased from Calbiotech (El Cajon, California, USA). The absorbance of calibrators and specimen was determined using an ELISA microplate reader, the multiskan ascent plate reader, purchased from MTX Lab Systems, Inc. (Bradenton, USA). The concentration was evaluated by means of a calibration curve established from the calibrators supplied with the kits.

### 2.7. Histological Analysis

The ovaries, uterus, and part of endometrial implant kept in 10% formalin were dehydrated in ethanol and embedded in paraffin. Paraffin-embedded tissues were cut (three different sections for ovary) at 5 *µ*m. After hematoxylin-eosin staining, the uterine and implant epithelial heights were assessed on a computer connected to a light microscope provided by Olympus (Tokyo, Japan). Ovarian follicles were counted two times at different periods, on three sections of the same ovary by the main investigator, and the final result for each ovary represented the mean of the two observations. Luteinized unruptured follicles (LUFs) were identified by the presence of oocytes not surrounded by cumulus oophorus cells within mature antral follicles [16, 21, 22].

### 2.8. Statistical Analysis

The GraphPad Prism 5.03 software was used to analyze data. Statistical significance and the difference among groups were evaluated by one-way analysis of variance (ANOVA) followed by the Tukey *post hoc* test for multiple comparisons. Differences were considered significant at *p* < 0.05. Data are presented as mean ± standard error of the mean (SEM).

## 3. Results

### 3.1. Effects of Treatments on the Variation of Endometrial Implant Volume and Tissue Levels of MDA and GSH

Table 1 shows that implant volume in [ENDO + DW] group increased by 104.706 ± 15.24 mm^3^. Following treatment, letrozole decreased implant volume by 82.68% as compared to the [ENDO + DW] group. The ethanol extract of avocado seeds induced a similar effect in a dose-dependent manner, as it decreased implant volume by 85, 118, and 150% at 12.5, 25, and 50 mg/kg, respectively, in comparison with the [ENDO + DW] group.

Tissue levels of MDA increased (*p* < 0.001) in all treated groups except in the [ENDO + EE50] group, where MDA level was similar to that of the [ENDO + DW] group.

The activity of GSH increased (*p* < 0.01) in all treated groups when compared to the [ENDO + DW] group.

### 3.2. Effects of Treatments on Tissue Levels of Estradiol and Serum Levels of Estradiol and Progesterone

**Figure 1:**
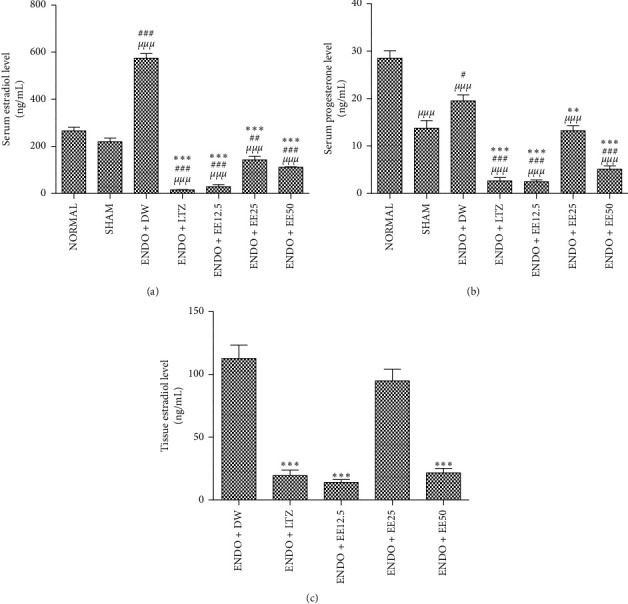
Effects of *P. americana* seeds on serum levels of estradiol (a) and progesterone (b) and tissue levels of estradiol (c). Data are presented as mean ± SEM, *n* = 5, ^*µµµ*^*p* < 0.001 versus [NORMAL], ^##^*p* < 0.01, ^###^*p* < 0.001 versus [SHAM], and ^*∗∗∗*^*p* < 0.001 versus [ENDO + DW]. SHAM: sham-operated animals; ENDO: animals with endometriosis; DW: distilled water; LTZ: letrozole; EE: ethanol extract of *P. americana* seeds.

Endometriosis increased serum levels of estradiol by 161% (*p* < 0.001). Letrozole and the ethanol extract of avocado seeds decreased this parameter by at least 75% (*p* < 0.001), as compared to the [ENDO + DW] group (Figure 1(a)).

Figure 1(b) shows that serum levels of progesterone increased by 42% (*p* < 0.001) in the [ENDO + DW] group, as compared to sham-operated animals. This parameter decreased by 86% (*p* < 0.001) following letrozole administration, in comparison with the [ENDO + DW] group. The ethanol extract of avocado seeds induced a similar effect by reducing serum levels of progesterone by 87, 32, and 74% (*p* < 0.01) at 12.5, 25, and 50 mg/kg, respectively, in comparison with the [ENDO + DW] group.

Tissue levels of estradiol decreased by at least 73% (*p* < 0.001) in all treated groups except in the [ENDO + EE25] group, where a 23% decrease of tissue levels of estradiol was observed, as compared to the [ENDO + DW] group (Figure 1(c)).

### 3.3. Effects of Treatments on Eutopic and Ectopic Endometrial Epithelial Heights

**Figure 2:**
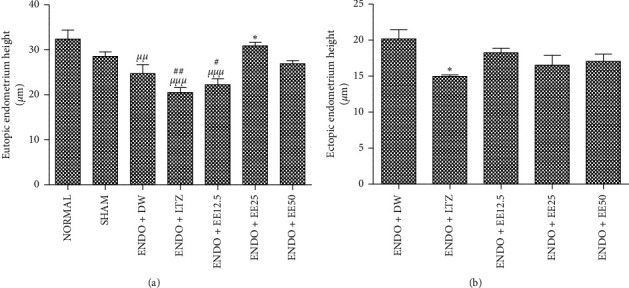
Eutopic (a) and ectopic (b) endometrial epithelial heights. Data are presented as mean ± SEM, *n* = 5, ^*µµ*^*p* < 0.01, ^*µµµ*^*p* < 0.001 versus [NORMAL], ^#^*p* < 0.05, ^##^*p* < 0.01 versus [SHAM], and ^*∗*^*p* < 0.05 versus [ENDO + DW]. SHAM: sham-operated animals; ENDO: animals with endometriosis; DW: distilled water; LTZ: letrozole; EE: ethanol extract of *P. americana* seeds.

Figure 2(a) shows that endometriosis decreased eutopic endometrial epithelial height by 13%, as compared to sham-operated animals, although this effect did not reach the level of statistical significance. The administration of letrozole further decreased (17% induction) the height of this organ, as compared to the [ENDO + DW] group. Following administration of the ethanol extract of avocado seeds, the endometrial epithelial height was almost similar to that of the [ENDO + DW] group, except in the [ENDO + EE 25] group, where a 25% (*p* < 0.05) increase in the height of this epithelium was observed, in comparison with the [ENDO + DW] group.

The administration of letrozole decreased the height of ectopic endometrium by 26% (*p* < 0.05), as compared to the [ENDO + DW] group (Figure 2(b)). The ethanol extract of avocado seeds also decreased the height of the ectopic endometrium in comparison with the [ENDO + DW] group at all tested doses, although this effect did not reach the level of statistical significance.

**Figure 3:**
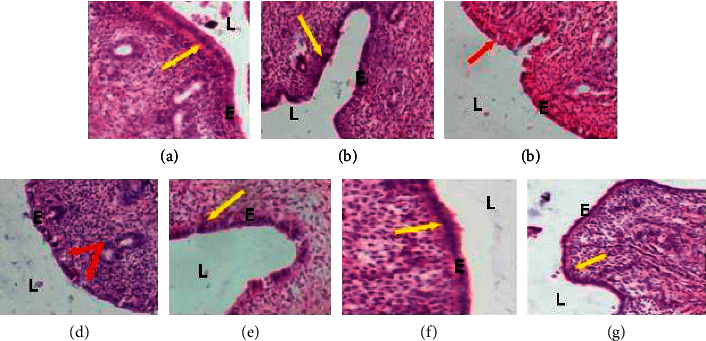
Microphotographs (×100, hematoxylin and eosin staining) of the uterus. *L* = lumen, *E* = epithelium, yellow *arrow* = mitosis, *red arrow* = necrosis. NORMAL: animals without surgery; SHAM: animals with hemi-ovariectomy; ENDO: animals with endometriosis; DW: distilled water; LTZ: letrozole; EE: ethanol extract of *P. americana* seeds. (a) NORMAL. (b) SHAM. (c) ENDO + DW. (d) ENDO + LTZ. (e) ENDO + EE12.5. (f) ENDO + EE25. (g) ENDO + EE50.

**Figure 4:**
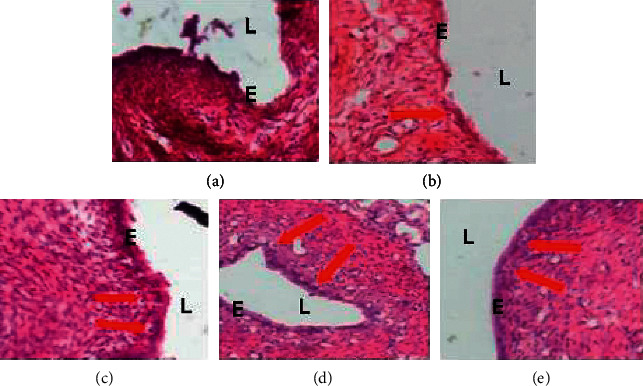
Microphotographs (×100, hematoxylin and eosin staining) of endometrial implants. *E* = epithelium, *L* = lumen, and red *arrows* = necrosis. ENDO: animals with endometriosis; DW: distilled water; LTZ: letrozole; EE: ethanol extract of *P. americana* seeds. (a) ENDO + DW. (b) ENDO + LTZ. (c) ENDO + EE12.5. (d) ENDO + EE25. (e) ENDO + EE50.

Microphotographs of the eutopic and ectopic endometrium of experimental animals are presented on Figures 3 and 4. On these figures, more necrosis figures are observed in both eutopic and ectopic endometrium in the [ENDO + DW] group. The same observation was done concerning these parameters in animals receiving letrozole. In animals treated with the ethanol extract of *P. americana* seeds, mitosis figures are observed on eutopic endometrium and necrosis figures on ectopic endometrium.

### 3.4. Effects of Treatments on Histological Score of the Ovaries

Table 2 shows that the number of antral follicles decreased by 71% (*p* < 0.05) in the ovaries of animals in the [ENDO + DW] group, as compared to sham-operated animals. Letrozole and the ethanol extract of avocado seeds increased the number of antral follicles by at least 2.5 times, as compared to the [ENDO + DW] group.

The number of corpora lutea in the [ENDO + DW] group decreased by 55% (*p* < 0.01), as compared to sham-operated animals. This parameter slightly increased following letrozole administration. The ethanol extract of avocado seeds increased the number of corpora lutea in all tested doses, with a significant (*p* < 0.05) effect observed at 25 mg/kg (Table 2).

The number of luteinized unruptured follicles (LUFs) was 10 times (*p* < 0.001) more elevated in [ENDO + DW] animals, in comparison with sham-operated animals. Following letrozole administration, the number of LUFs was similar to that of the [ENDO + DW] group. In contrast, the ethanol extract of avocado seeds decreased this parameter by at least 65% at all tested doses, as compared to the [ENDO + DW] group (Table 2).

**Figure 5:**
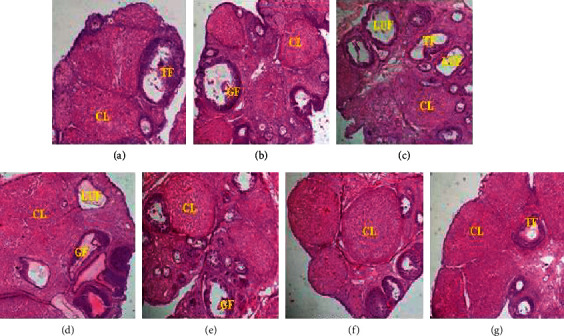
Microphotographs (×100, hematoxylin and eosin staining) of experimental rat ovaries. CL: corpora lutea; GF: Graafian follicle; LUF: luteinized unruptured follicle; TF: tertiary follicle. . (a) NORMAL. (b) SHAM. (c) ENDO + DW. (d) ENDO + LTZ. (e) ENDO + EE12.5. (f) ENDO + EE25. (g) ENDO + EE50.

Figure 5 shows microphotographs of the ovaries of experimental animals where the following follicles are identified: tertiary follicles, Graafian follicles, corpora lutea, and luteinized unruptured follicles.

## 4. Discussion

Endometriosis is a hormone-dependent disease in which products from macrophages such as growth factors, cytokines, and free oxygen radical induce endometriotic lesions proliferation [30, 31]. Indeed, the proliferative process observed in endometriosis is due to high estrogen levels, especially estradiol (E_2_) which was found to stimulate the growth of endometriosis lesions [32]. In this study, tissue levels of estradiol and serum levels of estradiol and progesterone were found elevated in the negative control (animals with endometriosis receiving distilled water). This high serum levels of progesterone were associated with elevated number of LUFs, as they were reported to produce the aforementioned hormone just like corpora lutea [33, 34], although their number was found reduced as compared to that observed in sham-operated animals. In addition, high tissue levels of estrogen confirm the resistance of ectopic cells to progesterone stimulation, as progesterone was found to stimulate the conversion of estradiol to estrone into the uterine tissue, resulting in the reduction of the level of estradiol and the decrease in its proliferative effect [32]. High serum levels of estradiol in the [ENDO + DW] group as compared to sham-operated animals could result from the hyperactivity of aromatase in the ectopic endometrium. In fact, an overexpression of this enzyme, as well as its hyperactivity, was reported to occur in endometriotic lesions [9], and may contribute to increasing serum levels of estradiol. These increased levels of estradiol may account for the enhanced endometriosis implant growth.

Following treatments, the ethanol extract of avocado seeds significantly (*p* < 0.001) decreased endometrial implant volume. This effect was associated with decreased estradiol levels in both serum and endometrial implants. Serum levels of progesterone also decreased, while tissue levels of MDA and GSH increased. These findings show that the extract of *P. americana* seeds induced a letrozole-like effect on endometrial implants and would have inhibited aromatase activity, resulting in decreased tissue estradiol levels leading to the regression of endometriosis implant volume. Furthermore, the decrease of this hormone probably facilitates lipid peroxidation, as estradiol was reported to be a cell survival factor [35] whose decrease causes oxidative stress which plays an important role in cell apoptosis [36, 37]. The increase in GSH levels suggests that the ethanol extract of *P. americana* seeds also acted by inhibiting the activity of Akt, thus protecting against further growth of endometriosis implants, as the activation of Akt is known to downregulate antioxidant defenses and to inhibit apoptosis, promoting tumor cell survival [37–39]. Results on serum levels of progesterone and estradiol also showed that the ethanol extract of *P. americana* seeds induced a nonlinear dose response also known as a biphasic or hormesis-type response [40, 41]. Hormesis was defined as a dose-response relationship phenomenon characterized by low-dose inhibition and high-dose stimulation or inversely, leading to a U-shaped, an inverse U-shaped, or a J-shaped dose response [41, 42]. This dose-response revolution is not yet fully understood.

Results obtained on ovarian follicles in the [ENDO + DW] group support the presence of a functional ectopic endometrium in these animals, as a result of the reported inhibitory effect of ectopic endometrium-released TIMPs on normal follicular development and ovulation [21, 43]. LUFs therefore indicate an impaired ovulation process and are thought to account for the endometriosis-related subfertility [21]. Following treatment, the ethanol extract of avocado seeds increased the number of antral follicles and corpora lutea and decreased that of LUFs, suggesting that this extract would have promoted ovarian follicle growth and maturation and stimulated ovulation. These results also support the observed reduction of endometriosis lesions, as the presence of a functional ectopic endometrium was reported to promote LUF formation by inhibiting ovulation [21]. However, results obtained on corpora lutea in the [ENDO + DW] group are different from our previous observations reporting increased number of corpora lutea in the ENDO control group [16, 22]. This may be due to the fact that animals in these studies were receiving estradiol or estradiol-like substances which would have enhanced the ovulatory process, despite the presence of endometriosis lesions.

On eutopic and ectopic epithelial heights, results showed that epithelial height of eutopic endometrium decreased while that of ectopic endometrium increased in the negative control (animals with endometriosis receiving distilled water). These results were associated with increased levels of serum estradiol and progesterone levels in these animals. Concerning the ectopic epithelial height, this result could be explained by the resistance of endometriotic cells to progesterone stimulation which normally acts by converting estradiol to estrone, to decrease estradiol-stimulation of cell growth [6]. The ethanol extract of *P. americana* seeds decreased epithelial height of ectopic endometrium probably because of decreased tissue estradiol levels. This hormone was reported to increase uterine epithelial height through an estrogen receptor alpha- (ER*α-*) mediated pathway [44]. On the eutopic endometrium, the ethanol extract of *P. americana* seeds increased epithelial height while the levels of estradiol were found lower than those of the negative control at the doses of 25 and 50 mg/kg. However, values of serum levels of estradiol obtained at these doses (112 ± 16.3 and 142 ± 1.5 ng/ml induced at 25 and 50 mg/kg, respectively) remained close to those of sham-operated animals (220 ± 16.00 ng/ml) and the normal control group (266 ± 16.00 ng/ml), but far to the level usually observed in ovariectomized rats (58 ± 8.27 ng/ml) [20]. These moderately high levels of estradiol may be responsible for cell hypertrophy observed on the uterine epithelium of animals treated with the ethanol extract of *P. americana* seeds.

## 5. Conclusion

Results of the current study showed that the ethanol extract of *P. americana* seeds displayed a potent inhibitory effect on the development of endometriotic implants through a mechanism involving the reduction of estradiol levels leading to ectopic cell damage. Therefore, the ethanol extract of *P. americana* seeds could be a potential alternative treatment for endometriosis.

## Figures and Tables

**Table 1:** Effects of the ethanol extract of *P. americana* seeds on the variation of endometriotic implant volume and tissue levels of MDA and GSH.

Groups	Variation of implant volume (mm^3^)	MDA (nM/mg)	GSH (nM/min/mg)
[ENDO + DW]	104.706 ± 15.24	5.102 ± 0.334	154.558 ± 13.529
[ENDO + LTZ]	24.005 ± 16.23^*∗*^	9.192 ± 0.551^*∗∗∗*^	305 ± 9.644^*∗∗∗*^
[ENDO + EE 12.5]	20.146 ± 11.22^*∗*^	9.307 ± 0.730^*∗∗∗*^	300.294 ± 14.670^*∗∗∗*^
[ENDO + EE 25]	(−) 24.907 ± 23.33^*∗∗∗*^	10.243 ± 0.408^*∗∗∗*^	344.411 ± 13.295^*∗∗∗*^
[ENDO + EE 50]	(−) 69.156 ± 13.82^*∗∗∗*^	5.256 + 0.393	233.529 ± 10.051^*∗∗*^

Results are expressed as mean ± SEM, *n* = 5, ^*∗*^*p* < 0.05, ^*∗∗*^*p* < 0.01, and ^*∗∗∗*^*p* < 0.001 versus [ENDO + DW]. ENDO: animals with endometriosis; DW: distilled water; LTZ: letrozole; EE: ethanol extract of *P. americana* seeds. MDA: malondialdehyde; GSH: glutathione.

**Table 2:** Effects of the ethanol extract of *P. americana* seeds on the number of antral follicles, luteinized unruptured follicles, and corpora lutea in rat ovaries.

Groups	Antral follicles	Corpora lutea	LUFs
[NORMAL]	15 ± 0.894	38.4 ± 2.785^*µµµ*^	0.2 ± 0.2
[SHAM]	5.6 ± 1.029^*µµµ*^	21 ± 2.588^*µµµ*^	0.4 ± 0.244
[ENDO + DW]	1.6 ± 0.509^*µµµ*#^	11.4 ± 1.568^*µµµ*##^	4 ± 0.316^*µµµ*###^
[ENDO + LTZ]	4.4 ± 0.509^*µµµ*^	15 ± 1.516^*µµµ*^	3.8 ± 0.583^*µµµ*###^
[ENDO + EE12.5]	4 ± 0.707^*µµµ*^	15.4 ± 1.288^*µµµ*^	0.6 ± 0.244^*∗∗∗*^
[ENDO + EE25]	4.6 ± 0.748^*µµµ*^	18.2 ± 1.2^*µµµ*^^*∗*^	1.4 ± 0.4^*∗∗∗*^
[ENDO + EE50]	4.2 ± 0.583^*µµµ*^	14.4 ± 2.111^*µµµ*^	0.8 ± 0.374^*∗∗∗*^

Results are expressed as mean ± SEM, *n* = 5, ^*µµµ*^*p* < 0.001 versus [NORMAL]; ^###^*p* < 0.001, ^##^*p* < 0.01, and ^#^*p* < 0.05, versus [SHAM]; ^*∗*^*p* < 0.05 and ^*∗∗∗*^*p* < 0.001 versus [ENDO + DW]. SHAM: sham-operated animals; ENDO: animals with endometriosis; DW: distilled water; LTZ: letrozole; EE: ethanol extract of *P. americana* seeds; LUFs: luteinized unruptured follicles.

## Data Availability

All the data used to support the findings of this study are available from the corresponding author upon reasonable request.
